# Lists of potentially inappropriate medications for older people in primary care: a systematic review of health outcomes

**DOI:** 10.1590/0102-311XEN016423

**Published:** 2024-05-20

**Authors:** Rafael Cardinali Rodrigues, Gabrielle Kéfrem Alves Gomes, Bárbara Manuella Cardoso Sodré, Rodrigo Fonseca Lima, Débora Santos Lula Barros, Ana Claudia Morais Godoy Figueiredo, Cristine Miron Stefani, Dayde Lane Mendonça da Silva

**Affiliations:** 1 Secretaria de Saúde do Distrito Federal, Brasília, Brasil.; 2 Universidade de Brasília, Brasília, Brasil.; 3 Escola Superior de Ciências da Saúde, Brasília, Brasil.

**Keywords:** Aged, Potentially Inappropriate Medication List, Primary Health Care, Idoso, Lista de Medicamentos Potencialmente Inapropriados, Atenção Primária à Saúde, Anciano, Lista de Medicamentos Potencialmente Inapropiados, Atención Primaria de Salud

## Abstract

This study is a systematic literature review of the association between lists of potentially inappropriate medications (PIM) in clinical practice and health outcomes of older adults followed up in primary health care. For this purpose, the PRISMA protocol was used to systematize the search for articles in the PubMed, Web of Science, Scopus, Cochrane Central, LIVIVO and LILACS databases, in addition to the gray literature. Studies with randomized clinical trials were selected, using explicit criteria (lists) for the identification and management of PIM in prescriptions of older patients in primary care. Of the 2,400 articles found, six were used for data extraction. The interventions resulted in significant reductions in the number of PIM and adverse drug events and, consequently, in potentially inappropriate prescriptions (PIP) in polymedicated older adults. However, there were no significant effects of the interventions on negative clinical outcomes, such as emergency room visits, hospitalizations and death, or on improving the health status of the older adults. The use of PIM lists promotes adequate medication prescriptions for older adults in primary health care, but further studies are needed to determine the impact of reducing PIM on primary clinical outcomes.

## Resumo

Este estudo revisou sistematicamente a literatura sobre a associação de listas de medicamentos potencialmente inapropriados (MPI) na prática clínica e desfechos de saúde na população idosa acompanhada na atenção primária à saúde. Para tanto, o protocolo PRISMA foi usado para sistematizar a busca de artigos nas bases de dados PubMed, Web of Science, Scopus, Cochrane Central, LIVIVO e LILACS, além da literatura cinzenta. Foram selecionados estudos com ensaios clínicos randomizados, incluindo a utilização de critérios explícitos (listas) para identificar e manejar MPI em prescrições para idosos atendidos na atenção primária. Dos 2.400 artigos encontrados, seis foram utilizados para extração de dados. As intervenções reduziram significativamente o número de MPI e eventos adversos a medicamentos e, consequentemente, nas prescrições potencialmente inadequadas em idosos polimedicados. No entanto, não houve efeitos significativos das intervenções sobre desfechos clínicos negativos (como visitas a serviços de emergência, hospitalizações e óbito) ou melhora das condições de saúde dos idosos. O uso de listas de MPI pode promover a adequação da prescrição de medicamentos para idosos na atenção primária à saúde, mas mais estudos são necessários para determinar os impactos da redução de MPI em desfechos clínicos primários.

## Resumen

Este estudio realizó una revisión sistemática en la literatura sobre la asociación de listas de medicamentos potencialmente inapropiados (MPI) en la práctica clínica y los resultados de salud en la población de edad avanzada monitoreada en atención primaria de salud. Para ello, se utilizó el protocolo PRISMA para sistematizar la búsqueda de artículos en las bases de datos PubMed, Web of Science, Scopus, Cochrane Central, LIVIVO y LILACS, además de la literatura gris. Se seleccionaron estudios con ensayos clínicos aleatorizados, incluyendo el uso de criterios explícitos (listas) para identificar y manejar MPI en prescripciones para adultos mayores atendidos en atención primaria. De los 2.400 artículos encontrados, seis se utilizaron para la recolección de datos. Las intervenciones tuvieron una significativa disminución en la cantidad de MPI y eventos adversos de medicamentos y, en consecuencia, en prescripciones potencialmente inapropiadas en adultos mayores polimedicados. Sin embargo, no hubo efectos significativos de las intervenciones en los resultados clínicos negativos (como consultas a servicios de urgencias, hospitalizaciones o muerte) o mejora en las condiciones de salud de los adultos mayores. El uso de listas de MPI puede promover una adecuada prescripción de medicamentos a los adultos mayores en la atención primaria de salud, si bien se necesitan más estudios para determinar los impactos de la reducción de MPI en los resultados clínicos primarios.

## Introduction

Medicines, which contribute decisively to the prevention and control of diseases and, consequently, to the improvement of the expectation and quality of life of the population, have become fundamental health technologies in the care process in contemporary times ^1^. In Brazil, 93% of older adults continuously use at least one medication for the treatment of chronic diseases, and 18% of this population use five or more medications, which is referred to in the literature as polypharmacy ^2^.

The global prevalence of polypharmacy is significantly higher in older adults aged 70 to 79 years (22%) and in those with four or more chronic diseases (60%) ^2^. In developed countries, the prevalence of polypharmacy varies from 39% to 45% in older adults ^3^. However, greater availability and access to medicines does not ensure safe and rational use of these technologies by older adults ^2^.

As described in the scientific literature, polypharmacy increases the likelihood of adverse drugs events (ADE), with a negative impact on health outcomes and investments in health interventions ^4^. In a 12-month study, Avery et al. ^5^ observed a medication error rate of 30.1% in patients taking five or more medications and of 47% in those taking ten or more medications. Although the prescription and use of multiple medications increases the risk of ADE, it is important to emphasize that assigning a numerical threshold is not sufficient to define the adequacy of drug treatments to the clinical conditions of users. Polypharmacy is often necessary and can be performed with quality, efficacy, and safety ^6^.

It is therefore essential that health professionals prioritize the quality of prescriptions in the care of the older adults, avoiding/correcting situations that contribute to the use of potentially inappropriate prescriptions (PIP) ^7^. To define PIP, explicit tools such as the Beers Criteria and the Screening Tool to Alert Doctors to the Right Treatment/Screening Tool of Older Persons (START/STOPP) can be used, as well as implicit tools based on judgments, such as the Medication Adequacy Index ^8^ ^,^ ^9^.

Considering that several studies have shown the association between PIP and ADE, lower rates of quality of life, increased hospital admissions and higher health care costs ^10^ ^,^ ^11^ ^,^ ^12^, this study aimed to carry out a systematic review of the literature to assess the following question: does the use of lists of potentially inappropriate medications (PIM) have an impact on the health outcomes of older adults monitored in primary health care (PHC)?

## Methodology

A systematic review was conducted by searching for studies in the following databases: PubMed, Embase (excluding MEDLINE), Cochrane Central (Trials), LIVIVO (excluding MEDLINE), Web of Science, Scopus, LILACS, ProQuest, OpenGrey, and Google Scholar (the first 100 results) in September 2020. The PRISMA Protocol guidelines were followed and this review was registered on the PROSPERO platform (n. CRD42020140090) and can be accessed at https://www.crd.york.ac.uk/prospero/#searchadvanced.

The terms used in the search are present in the *Medical Subject Headings* (MeSH), and their corresponding synonyms can be found in the *Health Sciences Descriptors* (DeCS, acronym in Portuguese). The full description of the terms used can be found in the Supplementary Material (Box S1; https://cadernos.ensp.fiocruz.br/static//arquivo/suppl-e00016423_9069.pdf). The search was not restricted by date of publication or by the language of the articles.

The PICOS strategy was used to structure the methodological process of this research. PICOS is an acronym for *Population/Patients*, *Intervention*, *Comparison/Control*, *Outcome*, and *Study design.* “P” corresponded to older patients: studies of people aged 65 years or over were included. “I” referred to the use of PIM lists. “C” referred to not using PIM lists. “O” included the health outcomes that were commonly found in this category: falls, hospitalization, visits to urgent/emergency services, and impact on quality of life. Lastly, “S” referred to clinical trials.

Titles and abstracts were analyzed by two independent and blinded evaluators. The search for articles was guided by the inclusion criteria: studies on older adults, adoption of the PIM list, research scenario in the PHC or in older adults receiving care in the community, longitudinal studies, and inclusion of health outcomes in the evaluation. Articles that used data from population surveys, private health insurance databases and private pharmacy databases were excluded. The agreement between them was analyzed using the kappa coefficient. Conflicts between the opinions of the two evaluators were adjudicated by a third evaluator, who also analyzed the cases blindly.

The data were extracted by four researchers considering the following variables: (i) author and year of publication; (ii) country in which the study was carried out; (iii) participants’ age; (iv) PIM lists used; (v) interventions performed in the intervention and control groups; (vi) main outcomes found; and (vii) main conclusion of the authors. The results were tabulated in an Excel spreadsheet (https://products.office.com/).

The included studies were organized in a Mendeley database (https://data.mendeley.com/) and on the Rayyan platform (https://rayyan.qcri.org). Bias analysis of the articles was performed using the *Critical Appraisal Tool*, from Joanna Briggs Institute (https://jbi.global/).

The GRADE system was used to classify the quality of evidence as very low (1 point), low (2 points), moderate (3 points) or high (≥ 4 points) according to the following criteria: risk of bias, inconsistency, indirect evidence, imprecision, publication bias, effect magnitude, dose-response gradient, and adjustment for confounders.

A random-effects meta-analysis was conducted using the DerSimonian and Laird method to estimate the summary odds ratio (OR) and respective 95% confidence intervals (95%CI) for interventions, protocol use and improvement of problems related to medications and inappropriate prescriptions. A statistical weight was assigned to each study according to the precision of confidence intervals. Statistical heterogeneity was estimated using I^2^, with values greater than 60% representing high statistical heterogeneity. Additional sensitivity, subgroup, and publication bias analyses were not conducted due to the small number of studies. Data analysis was conducted using Stata version 17 (https://www.stata.com).

## Results

**Figure 1:**
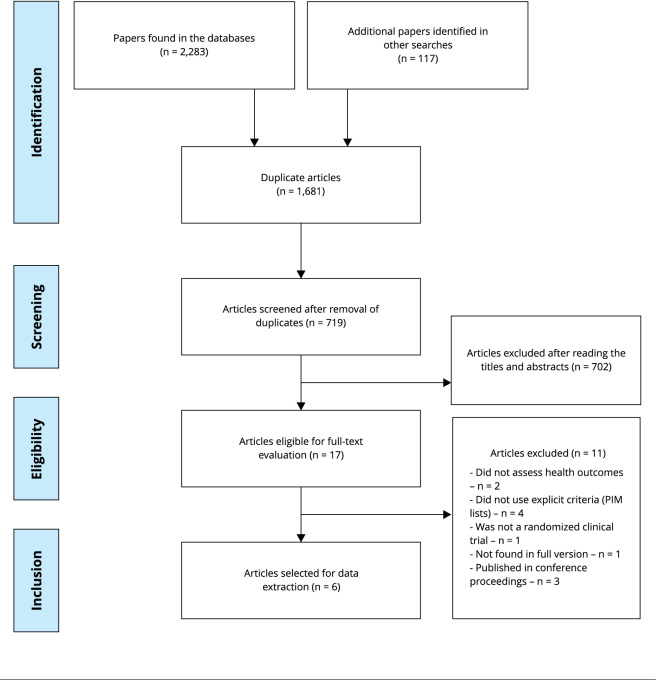
Flow diagram showing the selection process of articles for the systematic review.

In total, 2,400 studies were found. Of these 1,681 were excluded as they were duplicates, leaving 719 for the initial the analysis of titles and abstracts. As shown in Figure 1, 702 studies were not included for the following reasons: not including an older population (n = 107); being cross-sectional (n = 347), review (n = 58), qualitative (n = 19) or protocol (n = 36) studies; not using the MPI list (n = 73); or being conducted outside PHC (n = 79). At the end of this step, 17 studies were selected for full reading. The Kappa coefficient found was 0.993, indicating an almost perfect strength of agreement (according to the index by Landis & Koch ^13^).

PIM: potentially inappropriate medications.

In the eligibility phase, another 11 articles were excluded for the following reasons: not using the PIM list (n = 4); being published in conferences (n = 4); not available in full version (n = 1); and not assessing health outcomes (n = 2) (Supplementary Material, Box S2; https://cadernos.ensp.fiocruz.br/static//arquivo/suppl-e00016423_9069.pdf). In the end, six articles were eligible for discussion (Figure 1).

### Studies characteristics

The six selected articles, published in English, were obtained from four randomized clinical trials carried out in Europe, specifically in Spain, the Netherlands, Ireland, and Sweden. These studies used the START-STOPP list, the Beers Criteria and a specific list as explicit PIM criteria (OPTI-SCRIPT study) (Box 1) ^14^ ^,^ ^15^ ^,^ ^16^ ^,^ ^17^ ^,^ ^18^ ^,^ ^19^.

**Box 1:** Summary of the descriptive characteristics of the articles included (n = 6).

STUDY (YEAR)	COUNTRY	MEAN AGE IN YEARS	PIM LIST USED	GROUPS AND INTERVENTIONS	MAIN RESULTS AND OUTCOMES	MAIN CONCLUSIONS
Campins et al. ^14^ (2017)	Spain	Control: 78.78 (SD: 5.46), 57.4% women Intervention: 79.16 (SD: 5.50), 60.3% women	START-STOPP, version 2, 2015	Intervention (n = 252) Review of each participant’s pharmacotherapy by a clinical pharmacist, using the algorithm for GP-GP and the START-STOPP list to assess potentially inappropriate prescriptions. Presentation of pharmaceutical recommendations (discontinuing, including, replacing or changing the dose of the medication) and definition, together with each patient’s doctor, of the final recommendations. Agreement and implementation of recommendations after discussion between doctor and patient. Control (n = 251) Usual PHC	After 12 months, it was found that: (1) In the intervention group, 26.5% of prescriptions were classified as potentially inappropriate and 21.5% were optimized according to pharmaceutical recommendations (9.1% suspensions, 6.9% adjusted doses, 3.2% substitutions and 2.2% medication inclusions); (2) There were no significant differences between the intervention and control groups regarding the number of: emergency department visits (mean, SD): 0.9 (1.5) vs. 1.1 (1.5), p = 0.061; hospitalizations (n, %): 57 (23.3) vs. 63 (25.2), p = 0.616; deaths (n, %): 7 (2.8) vs. 6 (2.4), p = 0.784	The pharmacotherapy review using the GP-GP algorithm and the START/STOPP list reduced the number of prescribed drugs and improved the prescription appropriateness profile, but did not reduce emergency room visits, hospitalizations, and death in polymedicated (≥ 8 medications) older adults (≥ 70 years old)
Campins et al. ^15^ (2019)	Spain	Intervention: 79.1 (SD: 5.4), 61.6% women Control: 78.7 (SD: 5.5), 57.9% women	START-STOPP, version 2, 2015	Intervention (n = 245) Review of each participant’s pharmacotherapy by a clinical pharmacist, using the algorithm for GP-GP and the START-STOPP list to assess potential inappropriate prescriptions. Presentation of pharmaceutical recommendations (discontinuing, including, replacing or changing the dose of the medication) and definition, together with each patient’s doctor, of the final recommendations. Agreement and implementation of recommendations after discussion between doctor and patient. Control (n = 245) Usual PHC	After 12 months, the following was found: (1) A significantly greater reduction in annual medication expenditure in the intervention group than in the control group (-14.3% vs. -7.7%, p = 0.041); (2) A reduction in annual medication expenditure of EUR 233.75/patient (95%CI: 169.83; 297.67) in the intervention group and EUR 169.40/patient (95%CI: 103.37; 235.43) in the control group, indicating an annual saving of EUR 64.30/patient attributable to the intervention; (3) An estimated return of EUR 2.38 per Euro invested in the intervention program	The study showed that the intervention (prescription review by a clinical pharmacist) for polymedicated (≥ 8 medications) older patients (≥ 70 years) followed-up in PHC resulted in an annual reduction of approximately 7% in medication expenditures, suggesting a possible return on investment for the intervention
Willeboordse et al. ^16^ (2017)	Netherlands	Intervention: 77.8 (SD: 7.7), 64.4% women Control: 77.8 (SD: 8.0), 65.4% women	START-STOPP, version 1, 2008	Intervention (n = 275) Data collection from electronic medical records in PHC, from the pharmacy and from the screening questionnaire sent to the participants. Review of pharmacotherapy by a group of experts, consisting of a physician or nurse and a clinical pharmacist, using an adapted and electronic version of the STRIP, which includes the START-STOPP criteria. Sending the pharmacotherapeutic care plan, defined by the group of specialists, to the PHC physician. Agreement and implementation of the care plan after discussion between doctor and patient. Implemented recommendations were reported electronically to the pharmacy. Control (n = 243) Usual PHC. Data collection from the electronic medical record in PHC, from the pharmacy and from the screening questionnaire sent to the participants, and pharmacotherapy review by the group of specialists, but the doctor and patient did not receive the results of the analysis	After 6 months: (1) There was a higher number (%) of resolved ADE in the intervention group than in the control group (regression coefficient B: 22.6, 95%CI: 14.1; 31.1, p < 0.001). (2) There was no significant difference between the control and intervention groups in terms of self-reported quality of life based on the SF-12 and EQ5D-3L questionnaires (p > 0.05). (3) There were no significant differences between the intervention and control groups in terms of resolution (OR = 0.99, 95%CI: 0.62; 1.57, p = 0.96) and perception of severity (OR = 1.09, 95%CI: 0.73; 1.63, p = 0.67) of the main geriatric syndromes	The pharmacotherapy review based on the STRIP method and carried out by a group of specialists, including a clinical pharmacist, increased the resolution of DRP in the intervention group, but did not influence the course of the main geriatric syndromes or the perception of quality of life in polymedicated older patients in PHC
Lenander et al. ^17^ (2014)	Sweden	Intervention: 79.0 (SD: 77.8; 80.2), 65.4% women Control: 79.7 (SD: 78.4; 81.1), 68.6% women	Beers (1997)	Intervention (n = 107) Questionnaire on medication use and DRP sent to participants. Analysis of responses and pharmacotherapy review by a certified clinical pharmacist, using the Beers Criteria (1997) and the model of pharmaceutical care by Strand et al. ^43^ to identify and classify DRP. Blind data analysis by another independent clinical pharmacist. Presentation of pharmaceutical recommendations to patients prior to physician consultation. After 12 months, the questionnaire was sent back to the participants for comparison with the pre-intervention period. Control (n = 102) Submission of the questionnaire on medication use at baseline and after 12 months. Usual in PHC	After 12 months, the following was found: (1) A significant reduction in the number of DRP per patient in the intervention group, from 1.73 (95%CI: 1.42; 2.05) at baseline to 1.31 (95%CI: 1.02; 1.59) 6 months after the intervention, p = 0.02. (2) A significant reduction in the number of medications in the intervention group (from 8.6 to 7.9, p < 0.05), but not in the control group (from 7.4 to 7.5). (3) The mean number of hospital admissions was higher in the control group than in the intervention group (mean: 2.7 vs. 1.7; median: 2 vs. 1), as was the length of stay (mean: 18 vs. 12 days; median: 1.25 vs. 6 days); however, no significant differences were observed between the intervention and control groups. (4) Self-rated general health (scale from 1 to 5) remained unchanged in the intervention group, while in the control group there was a decrease in the score (p < 0.02), resulting in a significant difference between the groups, p = 0.047	The structured pharmacotherapy review performed by a qualified pharmacist helps to reduce the number of medications and prevent the decline in self-rated health in polymedicated (≥ 5 medications) older adults (≥ 65 years old) monitored in PHC
Clyne et al. ^18^ (2015)	Ireland	Intervention: 77.1 (SD: 4.9), 55.6% men Control: 76.4 (SD: 4.8), 51.5% men	OPTI-SCRIPT study with a list of potentially inappropriate drugs based on STOPP criteria	Intervention (n = 99) Academic detailing in 30-minute sessions between a clinical pharmacist and a general practitioner to review the pharmacotherapy of the patients included in the study. Prescription analyses were performed using a database with treatment algorithms containing evidence-based alternatives to PIM and PIP. Preparation of specific pamphlets (tailor-made) for patients with information on the PIM identified in the prescriptions. Control (n = 97) Usual PHC	After 12 months, the following was found: (1) A lower number (%) of patients with PIP in the intervention group than in the control group (52% vs. 77%), confirmed by relative risk (OR = 0.32, 95%CI: 0.15; 0.70, p = 0.02). (2) A lower number (mean) of PIP per patient in the intervention group than in the control group (0.70 vs. 1.18), with an incidence rate = 0.71 (95%CI: 0.50; 1.02), p = 0.49. (3) No significant difference in the WBQ-12 results between the intervention and control groups (23.6 vs. 24.0, mean: 0.41, 95%CI: -0.80; 1.07, p = 0.99)	The intervention of the OPTI-SCRIPT study reduced the number of PIP, mainly with proton pump inhibitors, but did not influence the beliefs about the medications or the perception of well-being of the older adults followed in PHC
Gillespie et al. ^19^ (2017)	Ireland	Intervention: 77.1 (SD: 4.9), 55.6% men Control: 76.4 (SD: 4.8), 51.5% men	OPTI-SCRIPT study with a list of potentially inappropriate drugs based on STOPP criteria	Intervention (n = 99) Academic detailing, in 30-minute sessions, between a clinical pharmacist and a general practitioner to review the pharmacotherapy of the patients included in the study. Prescription analyses were carried out using a database with treatment algorithms containing evidence-based alternatives to PIM and PIP. Preparation of specific pamphlets (tailor-made) for patients with information on the PIM identified in the prescriptions. Control (n = 97) Usual care	After 12 months, the following was found: (1) A non-significant increase in mean health care costs in the intervention group compared to the control group: EUR 3,075 (95%CI: 2,704; 3,446) vs. EUR 2,668 (95%CI: 2,297; 3,040). (2) A significant reduction in mean PIP in the intervention group compared to the control group: EUR 0.627 (95%CI: 0.588; 0.666) vs. EUR 1.006 (95%CI: 0.967; 1.045). (3) A nonsignificant increase in mean QALYs in the intervention group compared to the control group: EUR 0.671 (95%CI: 0.625; 0.716) vs. EUR 0.657 (95%CI: 0.612; 0.703). (4) An ICER per PIP averted of EUR 1,269 (95%CI: -1,400; 6,302) and an ICER per QALY gained of EUR 30,535 (95%CI: -334,846; 289,498)	Although the OPTI-SCRIPT study intervention was effective in reducing PIP in PHC in Ireland, the results of this study highlight the uncertainty regarding the cost-effectiveness of implementing the intervention in the service

95%CI: 95% confidence interval; ADE: adverse drugs events; DRP: drug-related problems; GP-GP: good palliative practice in geriatrics; ICER: incremental cost-effectiveness ratio; OR: odds ratio; PHC: primary health care; PIM: potentially inappropriate medications; PIP: potentially inappropriate prescriptions; QALY: quality-adjusted life year; SD: standard deviation; SF-12: *12-Item Short-Form Health Survey*; START-STOPP: Screening Tool to Alert Doctors to the Right Treatment/Screening Tool of Older Persons; STRIP: Systematic Tool for Reducing Inappropriate Prescribing; WBQ-12: *Well-Being Questionnaire*.

In all studies, the intervention involved a pharmacotherapy review by a clinical pharmacist to adjust drug prescriptions, followed by the development of a care plan with recommendations for pharmacotherapeutic optimization. The pharmacotherapy reviews differed regarding methods and instruments used, but all employed explicit criteria for identifying PIM in older adults. Two clinical trials included other professionals - physicians and nurses - in the pharmacotherapy review ^16^ ^,^ ^18^. The OPTI-SCRIPT Study involved training PHC physicians in the identification and management of PIP ^18^. This study used a web-based database with treatment algorithms and alternatives for PIM and PIP to support the pharmacotherapy review ^18^. The follow-up period of the studies ranged from 6 to 12 months (Box 1).

In total, the selected studies included 733 participants (99 to 275 patients per study) in the intervention group and 693 participants (97 to 251 patients per study) in the control group. The older adults, who had a mean age (standard deviation - SD) of 77.1 (4.9) to 79.2 (5.5) years in the intervention group and 76.4 (4.8) to 79.8 (5.5) years in the control group, were mostly female and treated with polypharmacy (Box 1).

Based on the *Critical Appraisal Tool*, all data were classified as having a low risk of bias (Box 2).

**Box 2:**
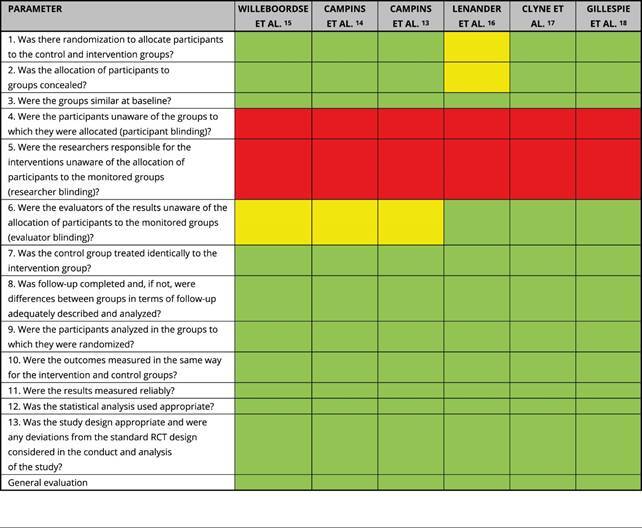
Risk of bias of the articles included in the systematic review.

RCT: randomized clinical trial. Note: red - high risk of bias, yellow - unknown risk of bias, and green - low risk of bias.

### Pharmacotherapy review and PIP

In the four randomized controlled trials, significant reductions in the mean number of medications and ADE per patient were observed, leading to the nonprescription of medications for the patients in the intervention groups and to the correction of PIP, as detailed as follows.

According to Campins et al. ^14^ ^,^ ^15^, the pharmacotherapy review carried out by a pharmacist based on the GP-GP (good palliative practice in geriatrics) algorithm and START-STOPP criteria (2015) significantly reduced the number of medications prescribed per patient after six months of follow-up (mean: 10.03 in the intervention group vs. 10.91 in the control group; p = 0.001) and the number of prescriptions per patient (mean [SD]: 109.1 [40.6], 95%CI: 104,0; 114,2 in the intervention group vs. 118.5 [43.1], 95%CI: 113.1; 123.9 in the control group; p = 0.013). In the intervention group, of the initial (baseline) medications, 9.1% were discontinued, 3.2% were substituted, and 6.9% were dose-adjusted. Of the final medications, 2.2% had been added after the intervention. After six months, the discontinuation and inclusion of new medications resulted in a 5% reduction in medications in the control group. The intervention also contributed to an increase in the adherence rate, which at baseline was 61.8% in the intervention group and 60.2% in the control group (p = 0.001). After six months, this rate increased to 76.4% in the intervention group and 64.1% in the control group (p = 0.005).

In the study by Lenander et al. ^17^, there was a significant reduction in the number of ADE per patient in the intervention group, from 1.73 (95%CI: 1.42; 2.05) at baseline to 1.31 (95%CI: 1.02; 1.59) after 12 months of follow-up (p = 0.02). This reduction was mainly due to the improvement in medication adherence in the intervention group (p = 0.048).

Willeboordse et al. ^16^ showed that, after six months, the pharmacotherapy review, carried out by a clinical pharmacist together with a physician or geriatric nurse, significantly reduced the percentage of ADEs in the intervention group (regression coefficient B: 22.6, 95%CI: 14.1; 31.1, p < 0.001).

According to Clyne et al. ^18^, after an intervention that included a pharmacotherapy review, carried out using a web database and patient information leaflets, participants in the intervention group had a lower number of PIP than patients in the control group (adjusted OR = 0.32, 95%CI: 0.15; 0.70, p = 0.02). The mean number of PIP (SD) in the intervention group was 0.70 (0.1), compared to 1.18 (0.1) in the control group (p = 0.02). However, when Poisson regression analysis was applied, the estimated number of PIP was 29% lower in the intervention group than in the control group, but this difference was not statistically significant (incidence rate = 0.71, 95%CI: 0.50; 1.02, p = 0.49).

### Clinical outcomes

Regarding health outcomes, three studies assessed the impact of interventions on hospitalizations and the use of emergency services. Campins et al. ^14^, after six months of follow-up, found no significant difference between the intervention and control groups regarding the mean number of admissions to the emergency room (mean [SD]: 0.9 [1.5] vs. 1.1 [1.5], p = 0.061) and the percentage of hospitalizations (number [%]: 57 [23.3] vs. 63 [25.2], p = 0.616). Similarly, after 12 months, Lenander et al. ^17^ found no significant difference between the intervention and control groups in terms of the number of hospitalizations.

Similarly, there was no difference in mortality between the control and intervention groups (n [%]: 6 [2.4] vs. 7 [2.8], p = 0.784) ^14^. The deaths that occurred in the intervention group were not related to changes in the patients’ pharmacotherapy ^14^.

As shown by Willeboordse et al. ^16^, no differences resulting from the pharmacotherapy review were found in the resolution or improvement of the main geriatric syndromes. In the intervention group, geriatric problems were resolved in 24.8% of cases, according to the self-perception of 44.7% of the patients. In the control group, there was an improvement in geriatric problems in 23% of cases, according to 41.5% of the older adults interviewed.

None of the studies found a significant increase in the quality of life reported by participants in the intervention groups. In the study by Campins et al. ^14^, the intervention made no difference in self-reported quality of life according to the EQL5D, which remained mostly stable in both groups at six months, with a change in baseline score (scale from 0 to 100) of -2.09 points in the intervention group and 0.67 points in the control group (p = 0.324). Willeboordse et al. ^16^, using different questionnaires, found no improvement in participants’ quality of life six months after the intervention (instrument: regression coefficient B [95%CI], p-value): EQ5D-3L: 0.01 [-0.02; 0.04], p = 0.53; EQ5D-3L VAS (0-100): 1.82, [-0.55; 4.18], p = 0.13; SF-12 MCS (0-100): -0.39 [-3.43; 2.65], p = 0.81; SF-12 PCS (0-100): -0.58 [-3.6; 2.53], p = 072.

Using the WBQ-12 well-being questionnaire (scale from 0 to 36), Clyne et al. ^17^ found no significant difference between the intervention and control groups at baseline (24.3 and 24.4, respectively) and at the end of the study (23.6 and 24.0, respectively) (adjusted OR = 0.41, 95%CI: -0.80; 1.07, p = 0.99).

On the other hand, Lenander et al. ^17^, when adapting a Likert scale (0 to 5 points) for the self-assessment of general health, found that self-reported health status remained unchanged in the intervention group one year after the start of the study (mean difference of 0.02, 95%CI: -0.15; 0.19). In the control group, however, there was a decrease in the overall score (mean difference of 0.27, 95%CI: 0.06; 0.48, p < 0.02), resulting in a significant difference in the perception of general health between the groups (p = 0.047).

### Economic evaluation

In an economic evaluation of a review of pharmacotherapy in older adults for the adequacy of PIP, Campins et al. ^15^ found that the reduction in annual expenditure with medication was EUR 233.75 per patient in the intervention group (95%CI: 169.83; 297.67) and EUR 169.40 per patient in the control group (95%CI: 103.37; 235.43). After 12 months of follow-up, the reduction in the individual percentage of expenditure was greater in the intervention group than in the control group (-14.3%, 95%CI: 19.4; 9.2 vs. -7.7%, 95%CI: 13.0; 2.35; p = 0.041). Considering the costs with human resources (pharmacist and physician fees) of implementing the interventions, an annual return of EUR 2.38 per patient (ranging from EUR 1.70 to EUR 3.40) was estimated for every EUR 1.00 invested in the pharmacotherapy review program.

Gillespie et al. ^19^ analyzed the cost-effectiveness of the OPTI-SCRIPT intervention for the adequacy of PIP in PHC. After 12 months of follow-up, the intervention was associated with a non-significant mean cost increase of EUR 407 (95%CI: -357; 1,170), a significant mean reduction of 0.379 in PIP (95%CI: 0.092; 0.666) and a non-significant mean increase of 0.013 in quality-adjusted life year (QALY) (95%CI: -0.016; 0.042). The incremental cost per PIP averted was EUR 1,269 (95%CI: -1,400; 6,302) and the incremental cost per QALY gained was EUR 30,535 (95%CI: -334,846; 289,498). The probability that the intervention was cost-effective was 0.602 at a threshold value of EUR 45,000 per QALY gained and at least 0.845 at a threshold value of EUR 2,500 or more per PIP averted.

### Data meta-analysis

**Figure 2:**
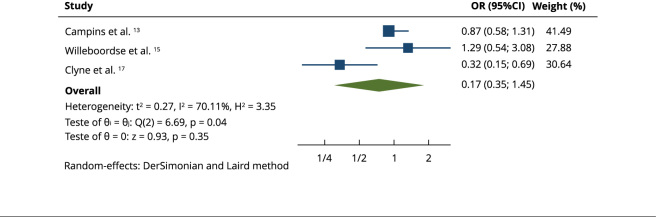
Data meta-analysis.

Only three studies had sufficient data to conduct a meta-analysis: Campins et al. ^14^; Willeboordse et al. ^16^ and Clyne et al. ^18^. Statistical heterogeneity was estimated to be 70.11% using I^2^ (Figure 2), with values above 60% representing high statistical heterogeneity. This fact was due to the studies presenting different statistical characteristics. The quality of evidence was rated as low according to the GRADE system (Box 3).

95%CI: 95% confidence interval; OR: odds ratio.

**Box 3:** Evaluation of the evidence.

PARTICIPANTS FOLLOWED-UP (STUDIES)	RISK OF BIAS	INCONSISTENCY	INDIRECT EVIDENCE	IMPRECISION	PUBLICATION BIAS	OVERALL CERTAINTY OF EVIDENCE
1,311 (3 randomized controlled trials)	High	High	Low	Low	Highly suspected publication bias. All potential confounders would reduce the demonstrated effect	⨁⨁◯◯ Low

Although the direction of the association measure indicated a trend toward benefit of the intervention (OR = 0.71), the results of the meta-analysis indicated that the intervention (use of protocols) did not cause a statistically significant difference (95%CI: 0.35; 1.45) between the intervention and control groups in relation to the outcome investigated (improvement in problems related to medications and inappropriate prescriptions). The results of the meta-analysis may have been influenced by the lack of studies on the subject.

## Discussion

Several studies have reported the association of polypharmacy with a greater likelihood of inappropriate drug use, adverse drug reactions, hospitalizations, admissions to emergency services, mortality, and other negative health outcomes ^20^ ^,^ ^21^ ^,^ ^22^ ^,^ ^23^ ^,^ ^24^. However, in this study, the use of the PIM list to review the pharmacotherapy of polymedicated older adults, followed by general practitioners in PHC, was not associated with improvement in clinical outcomes such as hospitalizations, major geriatric syndromes, death, and quality of life. The interventions also did not affect secondary outcomes such as user satisfaction with pharmacotherapy. Similarly, a Cochrane review failed to clarify whether the qualification of prescriptions was associated with positive health outcomes or improved quality of life ^25^. However, another study showed that pharmacotherapy reviews benefited health outcomes in a more complex group of patients, with more than five comorbidities per older person ^26^.

The level of acceptance of the interventions proposed by pharmacists directly reflects the ability to achieve positive outcomes; however, there is still a lot of resistance to the proposals. Willeboordse et al. ^16^ reported a rate of implementation of proposed interventions of only 47.8%. In a study carried out in São Paulo (Brazil) to evaluate clinical pharmacy services, the mean acceptance rate of interventions was 67.8% ^27^. Ignorance of the benefits of pharmaceutical interventions may be one of the reasons for the low acceptance rate.

In a systematic review, Thompson et al. ^28^ analyzed deprescribing tools and noted the complexity of this act. Scott et al. ^29^ confirmed this information, adding the possibility that this type of intervention takes a long time to implement. In this way, as the studies did not make it clear whether the prescribers had mastered the intervention tools, we cannot say that the lists were well applied.

Although OPT-SCRIPT has been shown to be significant in reducing PIPs, especially in relation to the use of proton pump inhibitors, the most recent studies that corroborate this information were carried out with institutionalized or hospitalized older adults and therefore cannot be compared with data from the older adults assisted in PHC ^30^ ^,^ ^31^ ^,^ ^32^.

The interventions used in the studies were not standardized. While some used pharmacotherapy reviews by pharmacists, others used electronic devices or broader health care teams in a shared care context. The tools used to measure quality of life also differed. A Cochrane systematic review states that when these variations occur, the impacts of pharmaceutical interventions may not be clearly defined ^25^.

There was also heterogeneity in the choice of PIM lists, which may have been reflected in the health outcomes. Although pharmacists performed the pharmacotherapy reviews in the studies investigated, different PIM lists were used. A systematic review cited 907 different drugs in the PIM lists analyzed ^33^. A study carried out in Ireland found PIM in 18.3% of patients using the Beers list, while this figure was 21.4% when the STOPP list was used ^34^. Another recent survey, conducted in Thailand, found even greater differences using the Winit-Watjana, Beers, and STOPP lists, with PIMs detected in 66.8%, 59% and 40.3%, respectively ^35^. Cooper et al. ^25^ suggested the development of a new tool with universal measures, easy to apply, and whose validity and reliability allow for the evaluation of the effectiveness of pharmaceutical interventions. It must be highlighted that this is a priority for future research, as the heterogeneity of medication lists can lead to different outcomes, making it difficult to compare results. Furthermore, medication management in older adults is extremely complex and evidence is still limited given the cultural and health care differences between countries ^25^.

Most studies indicated that significantly more pharmacotherapeutic problems were resolved in the intervention groups than in the control groups ^36^. And although the reduction in PIMs was not significant for improving health outcomes, given the mean analysis time, this information needs to be better investigated, considering that other studies show that inadequate prescriptions are associated with worse ADE rates, quality of life and visits to emergency services ^11^ ^,^ ^37^. Aguiar et al. ^38^ state that it is crucial to identify PIMs at risk of adverse cardiovascular events in the available lists, as this would allow for optimization of the prescribing process, with implications for clinical quality and treatment safety. In this sense, some lists already refer to cardiovascular ADEs such as myocardial infarction, attributing this outcome to the use of drugs such as cyclooxygenase-2 (COX-2) inhibitors ^9^ ^,^ ^39^ ^,^ ^40^ ^,^ ^41^.

Regarding costs with PIM, Gillespie et al. ^19^ found data on reduced expenses in the intervention group. There are a number of points to consider when addressing this issue. Although the costs of the professionals’ hourly work were estimated, work infrastructure costs, which could increase the indirect cost of the intervention, were not included. Furthermore, this study mentions a decrease in the prescription of new drugs, without strong therapeutic evidence, and an increase in the prescription of generic drugs, which would have a positive impact on the cost reduction outcome. It is also important to note that these expenses are only related to the purchase of unnecessary medications and do not include the costs of negative outcomes such as hospital expenses. These data are in line with a population-based study in Canada, which identified PIP expenditures of USD 419 million outside the hospital environment in 2013 ^42^.

A limitation of this study is that the search for articles was limited to the published scientific literature, that is, data from ongoing investigations were not included. On the other hand, this study used a comprehensive search process and a rigorous research strategy for the recruitment of scientific publications, enabling the selection of data that reflected the object under investigation.

## Conclusion

The pharmacotherapy reviews based on PIM lists led to a reduction in the number of PIM and ADE, and consequently in PIP among the older adults monitored in PHC. However, the qualification of prescriptions, observed in the intervention groups, did not affect negative clinical outcomes such as emergency room visits, hospitalizations and death, nor did it improve the health status of the older adults.

The aforementioned data indicate that the quality of the evidence is low, which means that caution must be exercised when interpreting it and using it in decision making. Therefore, there is a need for more robust clinical trials, including studies with larger sample sizes and longer follow-up, to provide solid evidence supporting a recommendation that is so widespread among specialists in the field of geriatrics and gerontology.

## References

[1] Wamble DE, Ciarametaro M, Dubois R (2019). The effect of medical technology innovations on patient outcomes,
1990-2015: results of a physician survey.. J Manag Care Spec Pharm.

[2] Ramos LR, Tavares NUL, Bertoldi AD, Farias MR, Oliveira MA, Luiza VL (2016). Polypharmacy and polymorbidity in older adults in Brazil a public
health challenge. Rev Saúde Pública.

[3] Charlesworth CJ, Smit E, Lee DSH, Alramadhan F, Odden MC (2015). Polypharmacy among adults aged 65 years and older in the United
States 1988-2010. J Gerontol A Biol Sci Med Sci.

[4] Masnoon N, Shakib S, Kalisch-Ellett L, Caughey GE (2017). What is polypharmacy A systematic review of
definitions. BMC Geriatr.

[5] Avery AJ, Ghaleb M, Barber N, Dean Franklin B, Armstrong SJ, Serumaga B (2013). The prevalence and nature of prescribing and monitoring errors in
English general practice a retrospective case note review. Br J Gen Pract.

[6] Oliveira PC, Silveira MR, Ceccato MGB, Reis AMM, Pinto IVL, Reis EA (2021). Prevalência e fatores associados à polifarmácia em idosos
atendidos na atenção primária à saúde em Belo Horizonte-MG,
Brasil. Ciênc Saúde Colet.

[7] Earl TR, Katapodis ND, Schneiderman SR, Shoemaker-Hunt SJ (2020). Using deprescribing practices and the screening tool of older
persons' potentially inappropriate prescriptions criteria to reduce harm and
preventable adverse drug events in older adults.. J Patient Saf.

[8] Fick DM, Semla TP, Steinman M, Brandt N, Dombrowski R, DuBeau CE (2015). American Geriatrics Society 2015 updated Beers Criteria for
potentially inappropriate medication use in older adults. J Am Geriatr Soc.

[9] O'Mahony D.O'Sullivan D.Byrne S.O'Connor MN.Ryan C.Gallagher
P (2015). STOPP/START criteria for potentially inappropriate prescribing in
older people version 2. Age Ageing.

[10] Fahrni ML, Azmy MT, Usir E, Aziz NA, Hassan Y (2019). Inappropriate prescribing defined by STOPP and START criteria and
its association with adverse drug events among hospitalized older patients a
multicentre, prospective study. PLoS One.

[11] Liew TM, Lee CS, Goh Shawn KL, Chang ZY (2019). Potentially inappropriate prescribing among older persons a
meta-analysis of observational studies. Ann Fam Med.

[12] Saqlain M, Ali H, Kamran S, Munir MU, Jahan S, Mazhar F (2020). Potentially inappropriate medications use and its association
with health-related quality of life among elderly cardiac
patients. Qual Life Res.

[13] Landis JR, Koch GG (1977). The measurement of observer agreement for categorical
data. Biometrics.

[14] Campins L, Serra-Prat M, Gózalo I, López D, Palomera E, Agustí C (2017). Randomized controlled trial of an intervention to improve drug
appropriateness in community dwelling polymedicated elderly
people. Fam Pract.

[15] Campins L, Serra-Prat M, Palomera E, Bolibar I, Martínez MÀ, Gallo P (2019). Reduction of pharmaceutical expenditure by a drug appropriateness
intervention in polymedicated elderly subjects in Catalonia
(Spain). Gac Sanit.

[16] Willeboordse F, Schellevis FG, Chau SH, Hugtenburg JG, Elders PJM (2017). The effectiveness of optimised clinical medication reviews for
geriatric patients Opti-Med a cluster randomised controlled
trial. Fam Pract.

[17] Lenander C, Elfsson B, Danielsson B, Midlöv P, Hasselström J (2014). Effects of a pharmacist-led structured medication review in
primary care on drug-related problems and hospital admission rates a
randomized controlled trial. Scand J Prim Health Care.

[18] Clyne B, Smith SM, Hughes CM, Boland F, Bradley MC, Cooper JA (2015). Effectiveness of a multifaceted intervention for potentially
inappropriate prescribing in older patients in primary care a
cluster-randomized controlled trial (OPTI-SCRIPT study). Ann Fam Med.

[19] Gillespie P, Clyne B, Raymakers A, Fahey T, Hughes CM, Smith SM (2017). Reducing potentially inappropriate prescribing for older people
in primary care cost-effectiveness of the OPTI-SCRIPT
intervention. Int J Technol Assess Health Care.

[20] Leelakanok N, Holcombe AL, Lund BC, Gu X, Schweizer ML (2017). Association between polypharmacy and death: a systematic review
and meta-analysis.. J Am Pharm Assoc (2003).

[21] Chang TI, Park H, Kim DW, Jeon EK, Rhee CM, Kalantar-Zadeh K (2020). Polypharmacy, hospitalization, and mortality risk a nationwide
cohort study. Sci Rep.

[22] Black CD, Thavorn K, Coyle D, Bjerre LM (2020). The health system costs of potentially inappropriate prescribing
a population-based, retrospective cohort study using linked health
administrative databases in Ontario, Canada. Pharmacoecon Open.

[23] Xing XX, Zhu C, Liang HY, Wang K, Chu YQ, Zhao LB (2019). Associations between potentially inappropriate medications and
adverse health outcomes in the elderly a systematic review and
meta-analysis. Ann Pharmacother.

[24] Agustín Sierra L, Rodríguez Salazar J, Jiménez-Muñoz AB, Molina Hernández MJ, Bermejo Bescós P, Iglesias Peinado I (2021). Potentially inappropriate medication in acute hospitalized
elderly patients with polypharmacy an observational study comparing PRISCUS,
STOPP, and Beers criteria. Eur J Clin Pharmacol.

[25] Cooper JA, Cadogan CA, Patterson SM, Kerse N, Bradley MC, Ryan C (2015). Interventions to improve the appropriate use of polypharmacy in
older people a Cochrane systematic review. BMJ Open.

[26] Leendertse AJ, de Koning GH, Goudswaard AN, Belitser SV, Verhoef M, de Gier HJ (2013). Preventing hospital admissions by reviewing medication (PHARM) in
primary care an open controlled study in an elderly
population. J Clin Pharm Ther.

[27] Melo DO, de Castro LLC (2017). Pharmacist's contribution to the promotion of access and rational
use of essential medicines in SUS. Ciênc Saúde Colet.

[28] Thompson W, Lundby C, Graabæk T, Nielsen DS, Ryg J, Søndergaard J (2019). Tools for deprescribing in frail older persons and those with
limited life expectancy a systematic review. J Am Geriatr Soc.

[29] Scott IA, Gray LC, Martin JH, Pillans PI, Mitchell CA (2013). Deciding when to stop towards evidence-based deprescribing of
drugs in older populations. Evid Based Med.

[30] Sharma R, Bansal P, Garg R, Ranjan R, Kumar R, Arora M (2020). Prevalence of potentially inappropriate medication and its
correlates in elderly hospitalized patients a cross-sectional study based on
Beers criteria. J Family Community Med.

[31] Liu Y, Zhu X, Li R, Zhang J, Zhang F (2020). Proton pump inhibitor utilisation and potentially inappropriate
prescribing analysis insights from a single-centred retrospective
study. BMJ Open.

[32] Debacq C, Bourgueil J, Aidoud A, Bleuet J, Mennecart M, Dardaine-Giraud V (2021). Persistence of effect of medication review on potentially
inappropriate prescriptions in older patients following hospital
discharge. Drugs Aging.

[33] Motter FR, Fritzen JS, Hilmer SN, Paniz EV, Vieira Paniz VM (2018). Potentially inappropriate medication in the elderly a systematic
review of validated explicit criteria. Eur J Clin Pharmacol.

[34] Ryan C, O'Mahony D, Kennedy J, Weedle P, Byrne S (2009). Potentially inappropriate prescribing in an Irish elderly
population in primary care.. Br J Clin Pharmacol.

[35] Vatcharavongvan P, Puttawanchai V (2019). Potentially inappropriate medications among the elderly in
primary care in Thailand from three different sets of
criteria. Pharm Pract (Granada).

[36] Rosenthal M, Holmes E, Banahan B (2016). Making MTM implementable and sustainable in community pharmacy is
it time for a different game plan?. Res Soc Adm Pharm.

[37] Jeon HL, Park J, Han E, Kim DS (2018). Potentially inappropriate medication and
hospitalization/emergency department visits among the elderly in
Korea. Int J Qual Health Care.

[38] Aguiar JP, Brito AM, Martins AP, Leufkens HGM, da Costa FA (2019). Potentially inappropriate medications with risk of cardiovascular
adverse events in the elderly a systematic review of tools addressing
inappropriate prescribing. J Clin Pharm Ther.

[39] Seo KW, Park JS, Tahk SJ, Shin JH (2017). A case of acute myocardial infarction induced by selective
cyclooxygenase-2 inhibitor. Chin Med J (Engl).

[40] Beers MH, Ouslander JG, Rollingher I, Reuben DB, Brooks J, Beck JC (1991). Explicit criteria for determining inappropriate medication use in
nursing home residents. Arch Intern Med.

[41] Renom-Guiteras A, Meyer G, Thürmann PA (2015). The EU(7)-PIM list a list of potentially inappropriate
medications for older people consented by experts from seven European
countries. Eur J Clin Pharmacol.

[42] Morgan SG, Hunt J, Rioux J, Proulx J, Weymann D, Tannenbaum C (2016). Frequency and cost of potentially inappropriate prescribing for
older adults a cross-sectional study. CMAJ Open.

[43] Strand LM, Morley PC, Cipolle RJ, Ramsey R, Lamsam GD (1990). Drug-related problems their structure and
function. DICP.

